# Effect of Delayed Initiation of Mepolizumab on EXACTO Scale Scores, Clinical Remission SEPAR-REMAS Criteria, and Functional Outcomes in Uncontrolled Severe Asthma: A Real-World Study

**DOI:** 10.3390/medsci14030387

**Published:** 2026-07-10

**Authors:** Antonio León-Lloreda, Belén Muñoz-Sánchez, María Luisa Polonio-González, David Carlos Echavarría-Kashmiri, Marta Ferrer-Galván, Auxiliadora Romero-Falcón, María Victoria Maestre-Sánchez, Juan Francisco Medina-Gallardo, Francisco Javier Álvarez-Gutiérrez

**Affiliations:** High-Complexity Asthma Unit, Medical–Surgical Unit of Respiratory Diseases, Hospital Universitario Virgen Del Rocío, Manuel Siurot, S/N, 41013 Seville, Spain; nonoleon97@gmail.com (A.L.-L.);

**Keywords:** severe eosinophilic asthma, mepolizumab, clinical remission, biologic timing, treatment delay

## Abstract

Introduction: Delayed initiation of mepolizumab may influence long-term disease control and the achievement of clinical and functional outcomes in patients with uncontrolled severe asthma (SUA), but no definitive conclusions have yet been established regarding the optimal timing for biologic initiation. The aim of this study was to evaluate, in a real-world clinical setting, the effect of delayed mepolizumab initiation—from the moment patients first met EMA eligibility criteria—on the treatment response (using the EXACTO scale), clinical remission (according to SEPAR-REMAS criteria) and lung function at 12 months and 3 years after treatment initiation. Material and Methods: We conducted a retrospective observational cohort study including 148 patients with SUA treated with mepolizumab from January 2017 to November 2024 in our hospital. Patients were stratified into tertiles according to delay: ≤5 months, 6–19 months, and >19 months. Baseline demographic, clinical, and lung function characteristics were analyzed. Results: Patients with shorter delay exhibited distinct significance baseline profiles, including higher eosinophil counts, lower BMI and current smoker, and better pre-treatment lung function (*p* < 0.05). Shorter delay was significantly associated with higher rates of good/complete response according to the EXACTO scale at both 12 months and 3 years (*p* < 0.05). Clinical remission rates were numerically higher in the early-treatment group, although differences did not reach statistical significance. No significant differences in lung function outcomes were observed between delay groups at either 12 months or 3 years. Conclusions: In conclusion, earlier initiation of mepolizumab after meeting EMA criteria is associated with improved clinical response, although it does not significantly influence remission rates or lung function recovery. These findings underscore the importance of timely treatment initiation and reinforce the relevance of accurate phenotypic and endotypic characterization to optimize biologic selection in SUA.

## 1. Introduction

Severe uncontrolled asthma (SUA) is defined, according to the Spanish Guideline for Asthma Management, by persistently poor symptom control, frequent or severe exacerbations, and airflow limitation despite high-intensity treatment [1]. Biologic therapies targeting type 2 inflammatory pathways, such as mepolizumab—an anti-interleukin 5 monoclonal antibody—have transformed the management of severe eosinophilic asthma by reducing exacerbations and oral corticosteroid (OCS) use, while improving clinical control and lung function [2]. The Spanish Society of Pulmonology and Thoracic Surgery (SEPAR) proposed the REMAS criteria as a multidimensional definition of clinical remission, requiring sustained fulfilment for ≥12 months of the following components: absence of exacerbations and rescue medication use, absence of OCS treatment, well-controlled asthma (defined as an ACT score ≥ 20), and normal or near-normal lung function (post-bronchodilator FEV_1_ ≥ 80% predicted or ≥90% of the patient’s best recorded value, together with a negative bronchodilator response) [3]. In addition, the 2025 Severe Asthma Consensus Document proposed the EXACTO scale as a tool to assess response to biologic treatment from 6 months onward and subsequently on an annual basis. Response is classified as absent, partial, good, or complete according to a score based on clinical, functional, and OCS reduction variables, with complete response defined as a score of 7 points (or 10 in OCS-dependent patients) and good response as 5–6 points (or 7–9 in OCS-dependent patients) [4]. Nevertheless, although the SEPAR-REMAS criteria and the EXACTO scale are not formally validated scales, both are expert consensus-based clinical assessment tools specifically designed for evaluating response to biologic therapy in severe asthma. Their use reflects current standard clinical practice and real-world decision-making regarding treatment continuation, optimization, or withdrawal.

Current evidence suggests that initiating mepolizumab during earlier stages of asthma or in patients with shorter disease duration is associated with higher rates of clinical remission and greater improvement in lung function [5,6,7]. However, there is limited evidence evaluating the effect of delayed initiation of mepolizumab according to the time elapsed since patients first fulfilled the European Medicines Agency (EMA) criteria for treatment initiation, as well as its relationship with treatment response assessed using specific tools for biologic-treated SUA patients, such as the EXACTO scale or SEPAR-REMAS clinical remission criteria in real world clinical practice. Therefore, the aim of this study was to evaluate, in a real-world clinical setting, the effect of delayed mepolizumab initiation—from the moment patients first met EMA eligibility criteria—on clinical remission (according to SEPAR-REMAS), treatment response (using the EXACTO scale), and lung function at 12 months and 3 years after treatment initiation.

## 2. Material and Methods

We conducted a retrospective observational cohort study including all patients aged ≥14 years with SUA treated with mepolizumab at the Severe Asthma Unit of our centre between January 2017 and November 2024. Baseline data collected at treatment initiation included demographic characteristics, clinical variables, OCS use, and pulmonary function parameters. All patients received fixed dose mepolizumab 100 mg administered subcutaneously every four weeks, with treatment adherence supervised by specialized nursing staff. Clinical remission (according to SEPAR-REMAS) and treatment response (according to EXACTO) were assessed at 12 months and 3 years following treatment initiation. Delay in treatment initiation was defined as the interval (in months) between the date on which patients first fulfilled EMA criteria for mepolizumab initiation (≥2 severe exacerbations or ≥1 hospitalization in the previous year and blood eosinophil count ≥ 150 cells/µL or historical eosinophil counts ≥ 300 cells/µL) [8] and the administration of the first dose. Patients were classified into tertiles after inspection of the distribution of treatment delay in order to obtain groups of similar size for comparison: ≤5 months (n = 56, 37.8%), 6–19 months (n = 43, 29.1%), and >19 months (n = 49, 33.1%), as this approach provided homogeneous and comparable groups. Data were obtained retrospectively from electronic medical records. Because of the retrospective observational design, all consecutive eligible patients treated with mepolizumab during the study period (January 2017–November 2024) were included. Categorical variables were compared using the chi-square test, whereas continuous variables were analyzed using the Kruskal–Wallis test, as data were not normally distributed. A two-sided *p* value < 0.05 was considered statistically significant. The study protocol was approved by the Provincial Clinical Research Ethics Committee of Seville (approval number 10/2025).

## 3. Results

148 patients were included in the analysis (mean age: 54 ± 14 years; 60% women), distributed into three groups according to delay in mepolizumab initiation: ≤5 months (37.8%), 6–19 months (29.1%), and >19 months (33.1%) (*p* = 0.424). Baseline characteristics at treatment initiation are shown in Table 1. Patients with shorter treatment delay (≤5 months) had significantly later asthma onset (*p* = 0.033), as well as older age at the time of biologic eligibility (*p* = 0.006). This subgroup also showed lower body mass index (*p* = 0.019), lower obesity prevalence (*p* = 0.005), and higher baseline eosinophilia (*p* = 0.037). Regarding pulmonary function prior to biologic initiation, this subgroup also exhibited higher FEV_1_ (%) values (*p* = 0.026) and higher absolute FEV_1_ in mL (*p* = 0.039). Additionally, patients with longer treatment delay (>19 months) had a higher proportion of active smokers (*p* = 0.002) and a lower proportion of biologic-naïve (*p* = 0.001).

**Figure 1:**
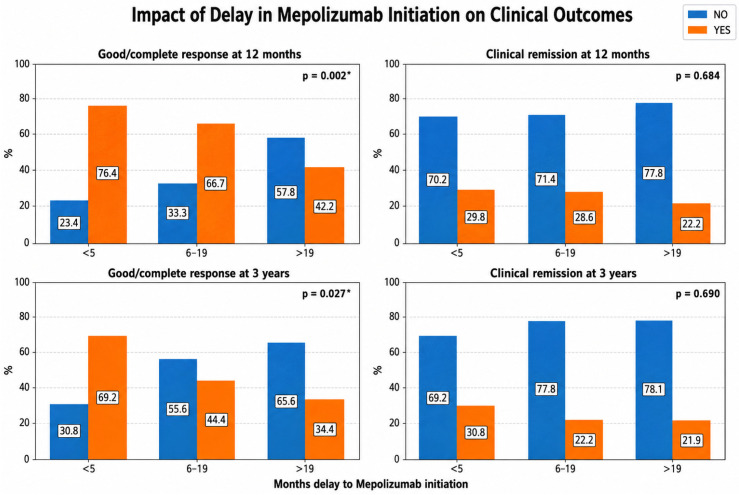
Impact of delay in Mepolizumab initiation on Clinical Outcomes. * Indicates a statistically significant difference between groups (*p* < 0.05). Statistical analysis employed (*p* < 0.05; 95% confidence interval [95% CI]): chi-square test.

**Figure 2:**
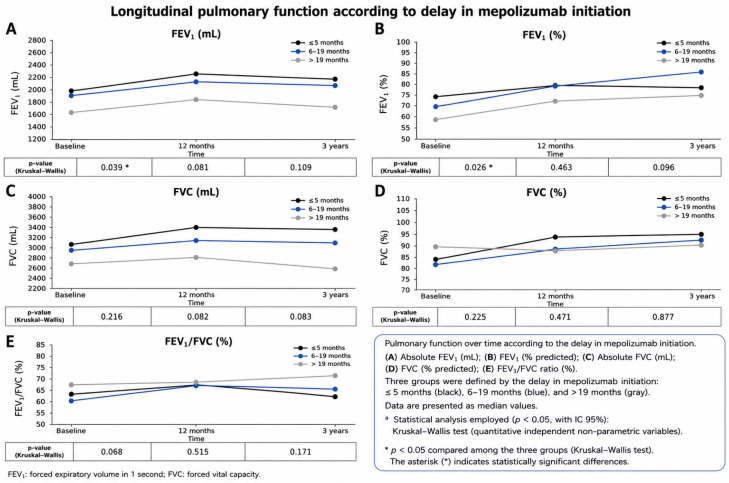
Longitudinal pulmonary function over time according to delay in mepolizumab initiation. ^a^ Statistical analysis employed (*p* < 0.05; 95% confidence interval [95% CI]): Kruskal–Wallis test for independent non-parametric quantitative variables. * *p* < 0.05 compared among the three groups (Kruskal–Wallis test). The asterisk (*) indicates statistically significant differences.

Figure 1 illustrates clinical outcomes following treatment initiation, where shorter delay groups showed significantly higher rates of good/complete response at both 12 months (*p* = 0.002) and 3 years (*p* = 0.027). However, as baseline characteristics significantly differed between delay groups (Table 1)—including age, BMI, obesity, smoking status, biologic-naïve status, eosinophilia, and pre-treatment lung function—a multivariable logistic regression analysis was performed to control for these potential confounders. The results of this adjusted model (Table 2) confirm that the time elapsed until mepolizumab initiation is an independent predictor of treatment success at both 12 months (OR 0.424; *p* = 0.002) and 3 years (OR 0.278; *p* = 0.001). Concerning clinical remission (according to SEPAR-REMAS), although remission was achieved more frequently in the shorter-delay group, no statistically significant differences were observed either at 12 months (*p* = 0.684) or at 3 years (*p* = 0.690). Finally, as it shows in Figure 2, lung function analysis at both 12 months and 3 years of follow-up showed no significant differences among treatment-delay groups.

## 4. Discussion

The potential impact of timing of biologic treatment initiation on short- and long-term clinical outcomes has been investigated in several immune-mediated inflammatory diseases, such as rheumatoid arthritis (RA), in which delayed introduction of biologic agents has been associated with structural joint damage and poorer clinical outcomes [9]. In asthma, however, the relevance of early biologic initiation remains an evolving area of research. Perez de Llano et al. draw a parallel between SUA and RA to analyze the potential benefits of establishing early treatment in SUA, concluding that a better clinical condition of the patient and more preserved lung function at the onset of biological treatment, together with a shorter duration of asthma, are associated with better response to biologics [6]. A post hoc analysis of the REDES study reported that longer asthma duration was associated with poorer baseline lung function and reduced functional recovery after 12 months of mepolizumab treatment, highlighting the potential importance of early treatment initiation [5]. Similarly, Pavord et al. observed higher rates of clinical remission among patients who initiated mepolizumab during earlier stages of the disease, characterized by shorter disease duration and/or lower severity [7]. In our study, although patients with longer treatment delay had significantly poorer lung function at baseline, total disease duration before biologic initiation was not specifically evaluated. Nevertheless, no definitive conclusions have yet been established regarding the optimal timing for biologic initiation or its impact on long-term clinical and functional evolution.

In our study, we considered that the timing for biologic initiation should be defined according to the date on which patients first fulfilled EMA eligibility criteria for mepolizumab, rather than solely according to disease duration or severity. This approach is supported, on the one hand, by the mechanism of action of the drug itself, targeting T2 inflammation in which eosinophils represent the main effector cell [2] thereby emphasizing the importance of accurate phenotypic and endotypic characterization of SUA patients and appropriate biologic selection. On the other hand, EMA criteria incorporate clinical indicators of poor asthma control, such as exacerbations and hospitalizations, thus enhancing their applicability in daily clinical practice. Therefore, from a practical and decision-making perspective, the relevant “time zero” for potential therapeutic intervention could be better defined by eligibility for biologic treatment rather than by disease onset, which may encompass heterogeneous phenotypes ranging from intermittent or mild asthma to later progression toward severe disease. This approach is particularly relevant in real-world studies conducted within healthcare systems where access to biologic therapies is strictly regulated according to EMA indications and hospital pharmacy protocols. In this context, clinicians’ capacity to intervene is effectively determined by the moment patients meet eligibility criteria, making this time point more clinically actionable than disease duration per se. Consequently, evaluating treatment delay from the moment of EMA eligibility provides a more standardized and practice-oriented framework to assess potential undertreatment or delayed access to biologic therapy.

Using this approach, we observed a significant effect on clinical disease evolution, as patients with longer delay exhibited poorer treatment response rates (according to the EXACTO scale) and lower clinical remission rates (according to SEPAR-REMAS), although the latter did not reach statistical significance. The absence of statistical significance for clinical remission may be explained by the strict definition of SEPAR-REMAS itself, since remission requires simultaneous fulfilment of all predefined components; therefore, failure to achieve a single variable prevents classification as remission despite an otherwise favourable clinical response. In contrast, functional evolution did not differ significantly between groups at either 12 months or 3 years. These findings suggest that delayed treatment initiation does not necessarily preclude functional recovery, although it may negatively affect clinical response to biologic therapy.

A possible hypothesis is a modulatory effect of mepolizumab on airway remodelling. The MESILICO and REMOMEPO studies suggest that anti–IL-5 therapy not only reduces eosinophilic inflammation but also attenuates structural airway changes [10,11]. REMOMEPO described reductions in reticular basement membrane thickness, bronchial smooth muscle mass, and extracellular matrix proteins, together with modulation of inflammatory cells and improvements in asthma control and FVC, whereas MESILICO reported similar structural and functional effects. These findings suggest that mepolizumab may facilitate functional recovery even in patients with poorer baseline lung function.

Another factor that may have influenced treatment response is asthma phenotype, particularly age at disease onset. Previous studies have shown that late-onset eosinophilic asthma is characterized by a distinct inflammatory profile and may exhibit greater responsiveness to biologic therapies targeting type 2 inflammation [12]. Unfortunately, age at asthma onset should be interpreted with the usual limitations of retrospective studies. Although an approximate age at disease onset can generally be obtained from the patient’s clinical history and medical records, it rarely corresponds to an objectively documented date and often relies, at least in part, on the patient’s recollection of when respiratory symptoms first appeared or when asthma was first diagnosed. Consequently, this variable is inherently subject to recall bias and potential misclassification, limiting its suitability for robust subgroup analyses. In our cohort, patients with shorter treatment delay tended to have a later age of asthma onset. Although this observation should be interpreted cautiously, it raises the possibility that differences in the underlying inflammatory phenotype may have contributed, at least in part, to the observed treatment response. Because late-onset asthma is more frequently associated with eosinophilic inflammation, these patients may represent a phenotype that is particularly suitable for anti–IL-5 therapy. However, this hypothesis cannot be confirmed with our data and should be explored in future prospective studies specifically designed to evaluate the interaction between treatment timing, asthma phenotype, and biologic response.

Altogether, these observations suggest the importance of appropriate patient selection based on inflammatory phenotype/endotype, comorbidities, and previous biologic exposure to achieve favourable clinical and functional outcomes in routine clinical practice.

Finally, several aspects may explain the differences between our findings and those of previous studies and constitute strengths of our work. These include the use of the EXACTO scale to assess treatment response and SEPAR-REMAS criteria to define clinical remission. Both tools integrate adequate clinical control, strict functional criteria (including bronchodilator testing and normal or near-normal FEV_1_), and absence of rescue medication use, thereby representing specific instruments designed for cohorts of biologic-treated SUA patients. Furthermore, the availability of follow-up data at both 12 months and 3 years provides one of the longest published evaluations of response to mepolizumab to date, since most available studies report follow-up periods of 12 or 24 months [5,6,7]. Additional strengths of our study include its independent design, absence of external pharmaceutical industry funding, and real-world clinical setting. Nevertheless, several limitations should be acknowledged. First, the retrospective single-center design may have introduced selection bias and reflects local referral pathways and treatment practices, which may limit the external validity and generalizability of our findings to other healthcare settings. Second, residual confounding cannot be completely excluded because of differences in baseline characteristics between treatment-delay groups. Third, the sample size may have limited the ability to detect differences in remission and lung function. Therefore, although multivariable adjustment strengthens our findings regarding the impact of treatment timing, the observational nature of this study warrants cautious interpretation of causality, our findings should be interpreted as hypothesis-generating and require confirmation in larger prospective multicenter studies.

In conclusion, in our real-world cohort of SUA patients treated with mepolizumab, longer delay in treatment initiation—from the time patients first fulfilled EMA eligibility criteria—was significantly associated with poorer treatment response according to the EXACTO scale, although without significantly compromising lung function recovery. These findings suggest that earlier initiation of mepolizumab after meeting EMA eligibility criteria may be associated with improved treatment response as assessed by the EXACTO scale. However, no significant differences were observed in clinical remission or lung function recovery. Therefore, these results should be interpreted cautiously and confirmed in prospective studies before firm conclusions regarding the optimal timing of biologic initiation can be established.

## Figures and Tables

**Table 1:** Baseline characteristics according to delay in mepolizumab initiation ^a^.

Variable	<5 Months N (SD/%)	6–19 Months N (SD/%)	>19 Months N (SD/%)	*p*-Value
Categorical variables, N (%)				
Patients	56 (37.8%)	43 (29.1%)	49 (33.1%)	0.424
Sex female	30 (53.6%)	26 (60.5%)	32 (65.3%)	0.468
Sex male	26 (46.4%)	17 (39.5%)	17 (34.7%)	
Obesity	11 (20.0%)	21 (48.8%)	20 (44.4%)	0.005 *
Current smoker	2 (3.6%)	3 (7.0%)	10 (20.4%)	0.002 *
Biologic-naïve patients	48 (85.7%)	39 (90.7%)	30 (61.2%)	0.001 *
Oral corticosteroid use (n)	18 (32.1%)	13 (30.2%)	19 (38.8%)	0.641
Hospital admissions (n)	13 (23.2%)	5 (11.6%)	5 (10.2%)	0.130
Rhinitis (n)	33 (58.9%)	29 (67.4%)	27 (55.1%)	0.470
Nasal polyposis (n)	20 (36.4%)	12 (27.9%)	16 (32.7%)	0.675
Chronic rhinosinusitis (n)	11 (19.6%)	7 (16.3%)	12 (24.5%)	0.634
Atopy (n)	26 (46.4%)	21 (48.8%)	26 (53.1%)	0.753
EGPA (n)	8 (14.3%)	3 (7.0%)	3 (6.1%)	0.291
Continuous variables, mean ± SD				
Age (years)	55.48 ± 15.17	51.23 ± 15.03	48.80 ± 12.91	0.022 *
Age at mepolizumab initiation (years)	56.91 ± 14.24	53.26 ± 15.31	53.71 ± 14.13	0.210
Age at mepolizumab eligibility (years)	56.75 ± 14.42	52.37 ± 15.42	48.73 ± 14.21	0.006 *
Age at asthma onset (years)	42.60 ± 18.41	39.56 ± 18.90	34.04 ± 18.53	0.033 *
BMI (kg/m^2^)	26.56 ± 5.25	29.83 ± 6.84	28.93 ± 5.41	0.019 *
Eosinophil count (cells/µL)	961.89 ± 927.91	726.51 ± 592.61	573.88 ± 256.18	0.037 *
Historical peak eosinophil count (cells/µL)	1236.07 ± 1087.64	1346.51 ± 1774.33	790.61 ± 326.08	0.183
Exacerbations (n)	4.77 ± 3.00	4.48 ± 2.75	5.67 ± 3.88	0.198
Oral corticosteroid dose (mg/day)	14.19 ± 9.74	13.62 ± 10.41	21.11 ± 13.95	0.138
Oral corticosteroid cycles (n)	4.41 ± 3.10	4.28 ± 2.81	4.41 ± 3.04	0.971
ACT score	12.25 ± 5.23	10.77 ± 4.93	11.00 ± 5.06	0.329
IgE (IU/mL)	326.91 ± 670.56	525.52 ± 1510.76	390.03 ± 694.86	0.466
Historical peak FeNO (ppb)	54.34 ± 48.68	61.69 ± 50.76	46.72 ± 37.85	0.366
FeNO (ppb)	52.87 ± 49.07	51.35 ± 44.94	50.07 ± 43.88	0.932
FEV1 baseline (%)	69.03 ± 20.02	76.19 ± 20.55	63.75 ± 20.18	0.026 *
FEV1 baseline (mL)	2010.89 ± 928.11	2045.58 ± 780.68	1692.86 ± 777.97	0.039 *
FVC baseline (%)	85.67 ± 18.34	97.17 ± 18.38	84.62 ± 18.81	0.225
FVC baseline (mL)	3035.71 ± 1132.64	2965.58 ± 976.18	2720.00 ± 932.50	0.216
FEV1/FVC baseline (%)	64.77 ± 13.81	68.71 ± 10.68	62.69 ± 13.30	0.068

^a^ Statistical analysis employed (*p* < 0.05, with IC 95%): Kruskall Wallis test (quantitative independent non-parametric variables) and Chi-squared (qualitative variables). The asterisk (*) indicates statistically significant differences.

**Table 2:** Independent association between treatment delay and favorable clinical response after multivariable logistic regression analysis ^a^.

Outcome	Independent Predictor	OR (95% CI)	*p*-Value	Nagelkerke R^2^
Good/complete EXACTO response at 12 months	Treatment delay (months)	0.424 (0.243–0.738)	0.002	0.134
Good/complete EXACTO response at 3 years	Treatment delay (months)	0.278 (0.128–0.607	0.001	0.346

Abbreviations: OR, odds ratio; CI, confidence interval. Odds ratios < 1 indicate that increasing treatment delay is associated with a lower probability of achieving a good or complete clinical response after adjustment for baseline clinical characteristics. ^a^ Statistical analysis employed (*p* < 0.05, with IC 95%): Binary multivariable logistic regression analysis was performed. The fit for the models was confirmed via the Omnibus test (12-month model: χ^2^ = 10.065; 3-year model: χ^2^ = 28.892). The final models demonstrated significant predictive power: Nagelkerke R^2^ of 0.134 at 12 months and 0.346 at 3 years.

## Data Availability

Regarding the Data Availability Statement, the data are not publicly available due to ethical and privacy restrictions. As these are clinical data, the Ethics Committee did not explicitly grant permission for public data sharing. Therefore, access to the data is restricted and can only be considered upon reasonable request and in accordance with applicable ethical requirements.

## Abbreviations

SUA: Severe uncontrolled asthma; SEPAR: Spanish Society of Pulmonology and Thoracic Surgery; OCS: oral corticosteroid; ACT: Asthma Control Test; EMA: European Medicines Agency; BMI: body mass index; EGPA: eosinophilic granulomatosis with polyangiitis; FEV1: forced expiratory volume in one second; FVC: forced vital capacity.
